# A Liquid Chromatography-Quadrupole-Time-of-Flight Mass Spectrometric Assay for the Quantification of Fabry Disease Biomarker Globotriaosylceramide (GB3) in Fabry Model Mouse

**DOI:** 10.3390/pharmaceutics10020069

**Published:** 2018-06-07

**Authors:** Seok-Ho Shin, Min-Ho Park, Jin-Ju Byeon, Byeong ill Lee, Yuri Park, Ah-ra Ko, Mi-ran Seong, Soyeon Lee, Mi Ra Kim, Jinwook Seo, Myung Eun Jung, Dong-Kyu Jin, Young G. Shin

**Affiliations:** 1 College of Pharmacy and Institute of Drug Research and Development, Chungnam National University, Daejeon 34134, Korea; seokho.shin.cnu@gmail.com (S.-H.S.); minho.park.cnu@gmail.com (M.-H.P.); jinju.byeon.cnu@gmail.com (J.-J.B.); byungill.lee.cnu@gmail.com (B.i.L.); yuri.park.cnu@gmail.com (Y.P.); 2 Research Institute for Future Medicine, Samsung Medical Center, Seoul 135-710, Korea; knoxgo@hanmail.net (A.-r.K.); miran.seong@sbri.co.kr (M.-r.-S.); soyeon86.lee@sbri.co.kr (S.L.); mira0411.kim@sbri.co.kr (M.R.K.); 3 Green Cross Research Center, Green Cross, Gyeonggi-do 446-850, Korea; jinugi1@greencross.com(J.S.); jungme@greencross.com (M.E.J.); 4 Department of Pediatrics, Samsung Medical Center, Sungkyunkwan University School of Medicine, Seoul 06351, Korea

**Keywords:** GB3, Fabry disease, LC-QTOF-MS/MS, B6;129-Gla^tm1Kul^/J

## Abstract

Fabry disease is a rare lysosomal storage disorder resulting from the lack of *α-Gal A* gene activity. Globotriaosylceramide (GB3, ceramide trihexoside) is a novel endogenous biomarker which predicts the incidence of Fabry disease. At the early stage efficacy/biomarker study, a rapid method to determine this biomarker in plasma and in all relevant tissues related to this disease simultaneously is required. However, the limited sample volume, as well as the various levels of GB3 in different matrices makes the GB3 quantitation very challenging. Hereby we developed a rapid method to identify GB3 in mouse plasma and various tissues. Preliminary stability tests were also performed in three different conditions: short-term, freeze-thaw, long-term. The calibration curve was well fitted over the concentration range of 0.042–10 μg/mL for GB3 in plasma and 0.082–20 μg/g for GB3 in various tissues. This method was successfully applied for the comparison of GB3 levels in Fabry model mice (B6;129-Gla^tm1Kul^/J), which has not been performed previously to the best of our knowledge.

## 1. Introduction

**Figure 1:**
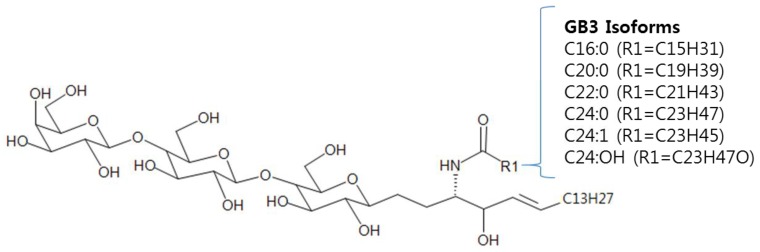
Structure of Globotriaosylceramide (GB3).

Fabry disease is an X-chromosome-linked inherited lysosomal storage disorder resulting from partial to total deficiency of the lysosomal enzyme α-galactosidase A due to the lack of *α-Gal A* gene activity [1,2,3]. As a result, glycosphingolipids, mainly Globotriaosylceramide (GB3) accumulates to the tissues primarily in the central nervous system, cardiovascular system, renal system (especially kidney), skin, and muscle [1,2,4,5]. The progression of the disease may affect the normal regulatory function of brain, heart, kidney, and other organs, leading to high morbidity and mortality, low quality of life [6,7]. Several methodologies on the treatment of Fabry disease has been developed including renal transplantation, somatic gene therapy, enzyme replacement using recombinant *α-Gal A* (e.g., Fabrazyme and Replagal) and the usage of small molecule pharmacological chaperone therapy (e.g., Migalastat HCl) [8,9,10,11,12,13]. With the usage of these treatments, GB3 levels in plasma and tissues dramatically decreases in Fabry patients [4,5,14,15,16]. These results suggest that GB3 can be used as a diagnostic biomarker and may be applied for the screening/monitoring in Fabry patients. However, the complexity of GB3 molecular structure (Figure 1) and a large number of possible GB3 isoforms made the GB3 analysis challenging [17,18].

Early assays performed with thin layer chromatography (TLC), gas chromatography (GC), and high-performance liquid chromatography (HPLC) were time consuming and several derivatization steps were required [18,19,20]. With the advent of electrospray ionization tandem mass spectrometry (ESI-MS/MS) for the quantification of GB3, certain derivatization steps are not required and faster, highly sensitive analysis was achievable [17,21,22]. Recently, a nano- Liquid chromatographic mass spectrometry (LC-MS/MS) method [23] and matrix-assisted laser desorption-ionization time-of-flight (MALDI-TOF) method [24] was also developed. Several sample preparation methods have also been introduced including solid phase extraction (SPE), protein precipitation (PPT), liquid-liquid extraction (LLE), and a combined liquid extraction/protein precipitation method [16,22,25,26,27,28].

Although these methods have been well applied for in vivo studies, a quick determination of GB3 from Fabry disease model is important particularly at the early stage research. In this study, we aimed on improving the analysis throughput without compromising the data quality with very limited sample volume. As a result, we used a simple protein-precipitation method without further steps discussed in the previous studies [18,19,20,26,27,28], using a small amount of plasma/tissue samples and we have improved the throughput significantly (>300 plasma and tissue samples per day) when compared to previous methods published in the literatures [18,19,20,26,27,28].

This method was well-qualified and applied for the quantification of GB3 in various organs, including plasma, heart, liver, spleen, kidney, and brain tissues to evaluate the correlation between GB3 level and Fabry disease progression. This method was also successfully applied to evaluate new drug candidates being developed in our lab by monitoring the GB3 level change in the Fabry model mice (B6;129-Gla^tm1Kul^/J) after dosing (data not shown).

## 2. Materials and Methods

### 2.1. Chemicals

GB3 and C17:0-GB3 were purchased from Matreya (Pleasant Gap, PA, USA). Several chemicals were obtained from Daejung Chemicals and Metals (Siheung, Gyonggi, Korea) of either GR or HPLC grade: formic acid, dimethyl sulfoxide (DMSO), methanol (MeOH), and distilled water. Bovine serum albumin was purchased from Sigma-Aldrich Chemical Corporation (St. Louis, MO, USA). Acetonitrile (ACN) from Avantor Performance Materials (3477 Corporate Parkway, Suite 200, Center Valley, PA, USA). Phosphate-buffered saline (PBS) was provided by Welgene (Dalseogu, Daegu, Korea).

### 2.2. Preparation of Stock Solution

GB3 stock solution was made at a concentration 1 mg/mL in DMSO. 0.2 mg/mL sub-stock solution was further prepared by spiking 0.2 mL of stock solution (1 mg/mL) into 0.8 mL of DMSO and both the stock and sub-stock solution were stored in the freezer at −20 °C. Working standard (STD) and quality control (QC) solutions were prepared by diluting the sub-stock solution. The internal standard (ISTD) *N*-heptadecanoyl ceramide trihexoside (C17:0-GB3) was made at 1 mg/mL in DMSO and stored in the refrigerator at −20 °C until use. The final ISTD spiking solution containing 1 μg/mL of C17:0-GB3 was prepared in methanol (MeOH).

### 2.3. Sample Preparation-Plasma

Blank Institute of Cancer Research (ICR) mouse plasma was diluted four-fold with 4% BSA-PBS for the preparation of calibration curve STD and QC samples. For example, 1 mL blank mouse plasma was added to 4 mL of 4% BSA-PBS for STD/QC samples. For the study samples, 10 μL aliquots of study sample was added to 40 μL of 4% BSA-PBS. Ten standard working solutions of GB3 were prepared freshly in duplicate by serially diluting the standard solution at a final concentration of 10, 5, 3.33, 2.50, 1.67, 1.11, 0.83, 0.37, 0.12, and 0.042 μg/mL using DMSO. A separate weighing of the GB3 reference standard was used to make QCs. Two QC working solutions of GB3 were prepared at a final concentration of 0.4 μg/mL medium QC (MQC) and 2 μg/mL high QC (HQC) using DMSO.

**Figure 2:**
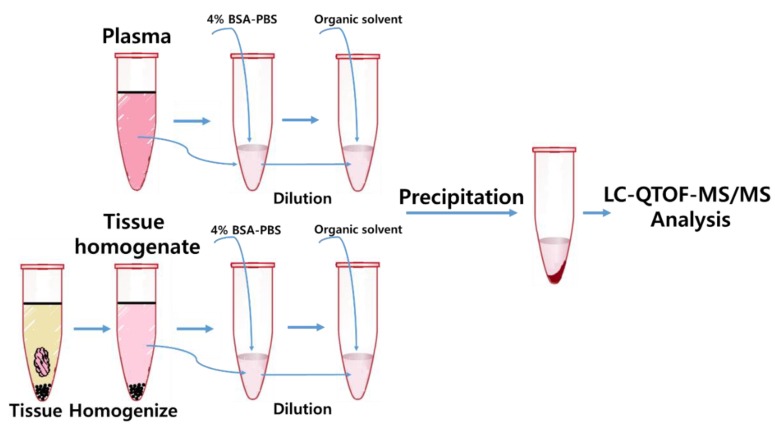
Sample preparation procedure.

Fifty microliters aliquot of the diluted plasma was added to cluster tubes. Five microliters of the corresponding standard working solution and QC solutions were also added to each cluster tubes, while 5 μL of make-up solution (DMSO) was spiked to study samples and blank samples (two blank samples with ISTD and two blank samples without ISTD (double blank)). For each sample, except for the double blanks, 200 μL of ISTD spike solution (1 μg/mL of C17:0-GB3 in MeOH) was added (200 μL of MeOH was added in the double blank as a make-up solution). All of the tubes were capped and vortexed for 1 min and centrifuged at 10,000 rpm for 5 min at 4 °C. After centrifuging, 185 μL of the supernatant was transferred into LC-vial for analysis. The schematic diagram of the plasma sample preparation procedure is demonstrated in Figure 2.

### 2.4. Sample Preparation–Tissues (Heart, Liver, Spleen, Kidney, Brain)

A four-fold volume of 4% BSA-PBS and several zirconia beads (2 mm diameter, Lysing Matrix I^®^, MP Biomedicals, Solon, OH, USA) was added to blank ICR mouse tissues and the study sample tissues and then homogenized using a FastPrep^®^-24 tissue homogenizer (MP Biomedicals, Solon, OH, USA) for 3 min to prepare tissue homogenates. Due to the high concentration of the GB3 levels in tissues, all tissue homogenates were re-diluted 200 times (500 times for spleen tissue homogenate) with 4% BSA-PBS. Eleven standard (ten standard for heart) working solutions of GB3 were prepared freshly in duplicate by serially diluting the standard solution at a final concentration of 20, 10, 6.67, 5, 3.33, 2.22, 1.67, 1.11, 0.74, 0.25, and 0.082 μg/mL using DMSO (ten points for heart: 20, 10, 6.67, 5, 3.33, 2.22, 1.67, 0.74, 0.25, and 0.082 μg/mL). A separate weighing of the GB3 reference standard was used to make QCs. Two QC working solutions of GB3 were prepared at a final concentration of 0.8 μg/mL medium QC (MQC) and 4 μg/mL high QC (HQC) using DMSO. Fifty microliter aliquots of each diluted tissue homogenates were added to cluster tubes. The same procedure used for plasma samples was applied for tissue samples and the schematic diagram of the tissue sample preparation procedure is also demonstrated in Figure 2.

### 2.5. Liquid Chromatographic Mass Spectrometry (LC-MS/MS) Condition

A Shimadzu CBM-20A HPLC pump controller (Shimadzu Corporation, Columbia, MD, USA), two Shimadzu LC-20AD pumps, a CTC HTS PAL autosampler (CEAP Technologies, Carrboro, NC, USA), and a quadrupole time-of-flight TripleTOF^TM^ 5600 mass spectrometer (Sciex, Foster City, CA, USA) equipped with a Duospray^TM^ ion source were used for the assay development. A Phenomenex^®^ Kinetex Phenyl-Hexyl column (2.1 × 50 mm, 2.6 μm) was used for the column separation. A binary gradient elution was employed using an aqueous mobile phase A, distilled and deionized water containing 0.1% formic acid; and an organic mobile phase B, acetonitrile containing 0.1% formic acid. The following gradient elution was used: from 0 min to 0.3 min, 35% B; from 0.3 min to 1.5 min by a linear gradient from 35% B to 95% B; 95% B was maintained for 0.8 min; and finally back to the initial condition (35% B) in 0.1 min and maintained for 1.6 min for re-equilibrium. The total run time was 4 min. The gradient was delivered at a flow rate of 0.5 mL/min and the injection volume was 10 μL. The single reaction monitoring with high sensitivity option (SRMHS) were recorded in positive ion mode. The most abundant ion was the sodium adduct [M + Na]^+^ ion for all GB3 isoforms. Since GB3 is a glycosphingolipid, it is composed of various degrees of saturation and oxidation form of fatty acids rather than being homogenous one [25]. Due to this heterogeneous composition, the most abundant six isoforms were selected to calculate the total GB3 concentration at mass to charge ratios (*m/z*) of: *m*/*z* 1046.7 (C16:0), *m*/*z* 1102.7 (C20:0), *m*/*z* 1130.8 (C22:0), *m*/*z* 1156.8 (C24:1), *m*/*z* 1158.8 (C24:0), and *m*/*z* 1174.8 (C24:0-OH). The GB3 ISTD (*N*-heptadecanoyl ceramide trihexoside (C17:0)) peak was observed at *m*/*z* 1060 (C17:0). The total area of six major isoforms was divided by the area of GB3 ISTD to achieve response ratio (Equation (1)). The mass spectrometer conditions are summarized in Table 1.

(1) $$\mathrm{Response}\;\mathrm{ratio}=\frac{\mathrm{Total}\;\mathrm{area}\;\mathrm{of}\;6\;\mathrm{major}\;\mathrm{GB}3\;\mathrm{isoforms}}{\mathrm{Area}\;\mathrm{of}\;\mathrm{GB}3\;\mathrm{ISTD}\;\left(C17:0\right)}$$

The source temperature was set to 500 °C with a curtain gas flow of 30 L/min. The ion spray voltage was set at 5500 V. The declustering potential was 100 V and the collision energy was 66 V. High-purity nitrogen gas was used for the nebulizer/Duospray^TM^ and the curtain gases. The mass spectrometer was automatically calibrated after the acquisition of every 20 samples using APCI positive calibration solution (Sciex, P/N 4460131) delivered by the calibration delivery system (CDS).

### 2.6. Method Qualification

A ‘fit-for-purpose’ approach was used for the method qualification. The qualification run contained duplicate calibration curves at ten concentrations for plasma and eleven concentrations for various tissues. The acceptance criteria for STDs and QCs in the qualification run were within ±30% of the precision and accuracy which is acceptable for the early stage drug discovery. Calibration was done by establishing a quadratic regression function, with an equation y = ax^2^ + bx + c after 1/concentration weighting. The accuracy was calculated at each QC concentration as the ratio of the measured concentration to the nominal concentration multiplied by 100%.

A preliminary stability test was conducted in mouse plasma and various tissue (heart, liver, spleen, kidney, and brain) samples under different conditions, such as short-term, long-term, and freeze-thaw stability using triplicates of high QC samples (2 μg/mL for plasma and 4 μg/mL for tissues). The short-term stability was determined at room temperature for 3 h. The long-term stability was determined after two weeks by analyzing QC samples kept frozen at −80 °C. For the freeze-thaw stability, the samples were subjected to three freeze and thaw cycles at −80 °C. The acceptance criteria for all stability tests were within ±30% of the precision and accuracy.

### 2.7. Software

Analyst^®^ TF Version 1.6 (Sciex, Foster City, CA, USA) operated with Windows^®^ (Microsoft Corp., Redmond, WA, USA) was used for the instrument control as well as the data acquisition. MultiQuant^®^ Version 2.1.1 (Sciex, Foster City, CA, USA) was used for the peak integration. Excel 2015 (Microsoft) was used for all calculations including peak area ratios, standard curve regressions, sample concentration values, and descriptive statistics.

### 2.8. Application for Animal Study

Fabry disease model mice, generated on a 129SVJ X C57BL/6 background by targeted disruption of the murine *α-Gal A* gene (*Gla*), have been used in various studies [29,30]. These B6;129-Gla^tm1Kul^/J (also known as *α-Gal A* KO) mice, purchased from the Jackson laboratory (JAX stock #003535, 600 Main Street, BH, ME USA), were maintained by brother X sister mating. Male KO mice used in this study were selected by genotyping using polymerase chain reaction (PCR) analysis in tail snip DNA. Wild-type mice with the ICR strain were used as controls throughout the studies. Mice were housed in groups of 2–5 per cage under standard housing conditions. Mice were given a standard rodent diet and housed in a pathogen-free animal facility.

Mice were distributed into three different groups (four mice per group; 10, 12, and 14 weeks of age) to measure the age-related GB3 level changes in KO and WT male mice. All the KO mice were compared to WT mice for the relative GB3 levels in plasma and each of the tissues. At the study completion point, each of the mice was euthanized with CO_2_. Whole blood was collected in a cluster tube and then centrifuged at 10,000 rpm for 5 min (4 °C). After centrifugation, the supernatant plasma layer was transferred to another cluster tube and stored in a deep freezer at −80 °C until analysis. Tissues (heart, liver, spleen, kidney, brain) were quickly removed, rinsed in cold phosphate-buffered saline (PBS) and stored in the deep freezer at −80 °C until analysis.

All procedures performed on the mice were reviewed and approved by the Institutional Animal Care and Use Committee (IACUC) of Samsung Biomedical Research Institute (Identification code: 20160216002, approval date: 9 March 2016) and were conducted in accord with guidelines established by the Association for Assessment and Accreditation of Laboratory Animal Care International (AAALAC International).

## 3. Results

### 3.1. Method Development: Sample Preparation and LC-MS/MS Analysis 

At the early stage of biomarker research, quick identification of the GB3 level from a small amount of plasma/tissue samples from Fabry disease model is important. In the previous studies, several time-consuming sample preparation methods have been reported, including sample enrichment, such as evaporation/reconstitution steps and purification steps (LLE, SPE) [16,22,25,26,27,28]. These methods took a lot of time for sample preparation and also require a large amount (>100 μL) of study samples. In addition, none of the previous studies were done for the GB3 level in the Fabry model mice (B6;129-Gla^tm1Kul^/J) to our knowledge in various tissues, including brains, which was developed by our own lab. Due to significantly different levels of GB3 and its analogs in plasma, as well as in various tissues, developing a method that can cover all these various study samples is very challenging. Therefore, we have focused on the development of a rapid sample preparation procedure with the usage of a less amount of plasma/tissue samples for the in vivo Fabry biomarker study. As a result, we developed a simple protein-precipitation method using minimum amount (10 μL) of samples without evaporation/reconstitution, derivatization steps or sample purification steps (solid phase extraction or liquid-liquid extraction). We also shortened the total chromatographic run time in LC-MS to 4 min and maximized sample analytical efficiency. Compared to the previous studies [18,19,20,26,27,28], our method met our throughput acceptance criteria (>300 plasma and tissue samples per day) using a small amount of study samples without further enrichment/purification for sample preparation procedure.

**Figure 3:**
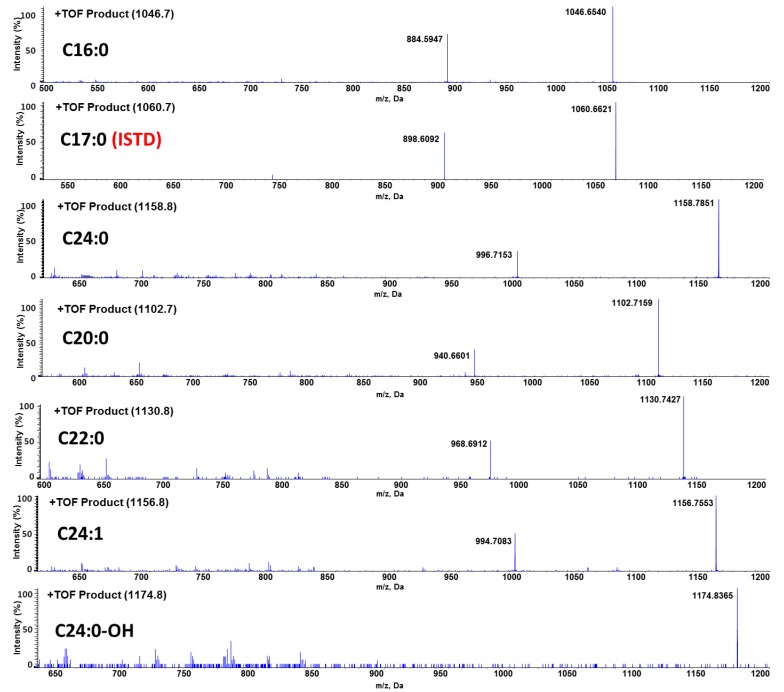
Product ion mass spectra of six GB3 isoforms and internal standard (ISTD).

**Figure 4:**
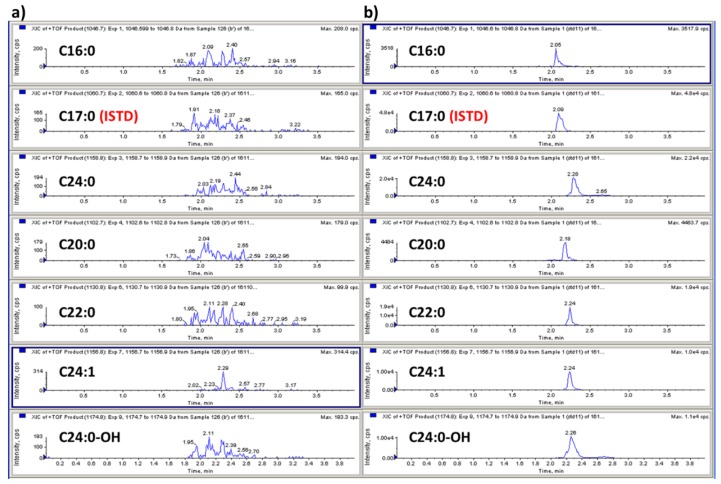
(**a**) LC-MS chromatograms of blank plasma, and (**b**) LC-MS chromatograms of GB3 spiked plasma.

A liquid chromatography–quadrupole time-of-flight mass spectrometric method (LC-QTOF-MS/MS) in the positive ion mode was used to identify GB3 major isoforms. The most abundant ion in the full scan mode was the sodium adduct [M + Na]^+^ ion for all GB3 isoforms. Due to the heterogeneous fatty acid components of GB3, the most abundant six isoforms were selected for the quantitation: *m*/*z* 1046.7 (C16:0), *m*/*z* 1102.7 (C20:0), *m*/*z* 1130.8 (C22:0), *m*/*z* 1156.8 (C24:1), *m*/*z* 1158.8 (C24:0), and *m*/*z* 1174.8 (C24:0-OH) (Figure 3). Each ion was detected using the single reaction monitoring with the high sensitivity (SRMHS) mode. The product ion showing the most abundant intensity was still the intact parent ion itself when the optimized collision energy was used. Due to the high resolution and accurate mass specificity of the TOF-MS the parent-to-parent ion transition was considered to be applicable [31], and the LC-MS chromatogram also showed good separation (Figure 4). Therefore, this parent-to-parent ion transition approach was used for the analysis of all GB3 isoforms in this study. Although the parent-daughter ion transition by the loss of 162 (except C24:0-OH) was not used for the quantitation of Gb3 isoforms, the unique transitions by the loss of 162 have been monitored as secondary transitions for the confirmation purpose of Gb3 isoforms. The broader peak width of C24:0-OH than those of other GB3 isoforms was likely due to the interaction between the hydroxyl group of C24:0-OH and the column stationary phase.

### 3.2. Method Qualification

#### 3.2.1. Calibration Curve, Accuracy, and Precision

**Figure 5:**
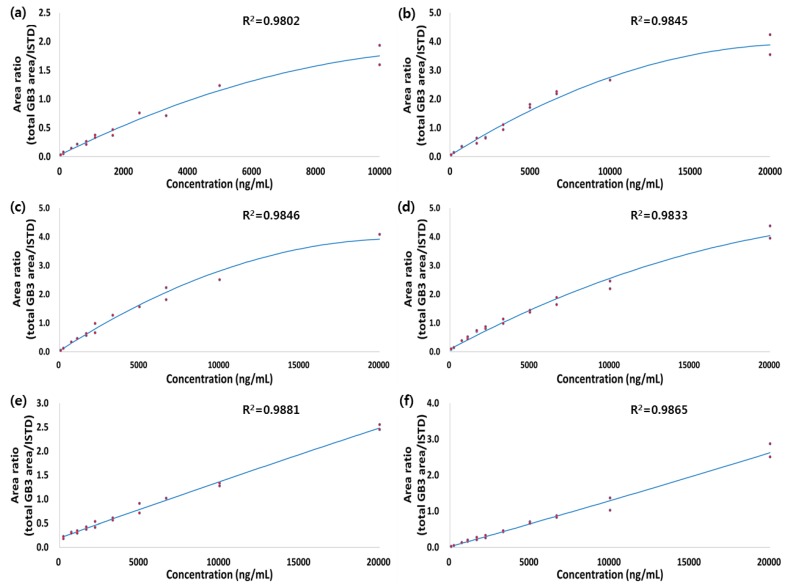
Calibration curve of GB3 in (**a**) plasma; (**b**) heart; (**c**) liver; (**d**) spleen; (**e**) kidney; and (**f**) brain.

Calibration curves with ten points (plasma, heart) and eleven points (liver, spleen, brain, kidney) were freshly prepared in duplicate for all datasets. The lower limit of quantification (LLOQ) of the assay was determined to be 0.042 μg/mL for plasma and 0.082 μg/g for all tissues based on the signal-to-noise ratio >5, respectively. The final concentration of the calibration curve range was 0.042–10 μg/mL for GB3 in plasma (10 levels) and 0.082–20 μg/g for GB3 in tissues (10 levels in heart and 11 levels in liver, spleen, kidney, and brain). One advantage of TOF mass spectrometer is the flexibility of MS data processing after acquisition. Therefore, based on the selectivity or sensitivity required for the experiment, various ion extraction width (e.g., 0.2 amu to 0.05 amu, or smaller) could be used to construct the flexible calibration curves, if needed. The quadratic regression of the curves for peak area ratios versus concentrations were weighted by 1/concentration. Calculated coefficient of determination (r) values for calibration curves were used to evaluate the fit of the curves. The correlation coefficient of the calibration curves for plasma and each of the tissues were ≥0.98 and are shown in Figure 5. Assay performance was determined by assessing precision (RSD (%)) and mean accuracy (%) of the QC samples and the results are shown in Table 2. Within-run accuracy and precision were evaluated using triplicates from each of the two QC concentrations. The within-run accuracy met the acceptance criteria of ±30%, ranging from 75 to 110% with precision values (RSD (%)) ≤ 30% for plasma and tissue samples, which is acceptable for an early stage biomarker study.

#### 3.2.2. Preliminary Stability

Stability assessments were carried out to demonstrate that GB3 (either its stock solution or the spiked samples) was stable under certain sample storage and processing conditions. All of the stability tests were conducted in mouse plasma and various tissues (heart, liver, spleen, kidney, and brain) samples using triplicates of high QC samples (2 μg/mL for plasma and 4 μg/mL for tissues).

Typically three levels of QC samples (low, medium, and high) are used for the full bioanalytical method validation. However, our method is not for the full bioanalytical method validation for the IND enabling study but for the fit-for-purpose qualification method of the Gb3 biomarker at the early discovery stage for the Fabry disease mouse model. The Fabry disease model mouse normally generates very high levels of Gb3 isoforms and, therefore, one level of QCs (high) would be sufficient to demonstrate the integrity of our stability experiment. Each of the stability results are shown in Table 3, Table 4 and Table 5.

The stability tests for plasma were performed using triplicate of QC samples at high QC (2 μg/mL) level. The peak area ratio between total GB3 (six isoforms) and ISTD was used for the stability evaluation. The preliminary stability test showed GB3 in plasma QC sample was stable for at least 3 h at room temperature and three freeze-thaw cycles and at least two weeks of stability while storage at −20 °C with a precision value (RSD (%)) within 30% and the mean accuracy within ±30%.

The stability tests for various tissue samples were also performed using triplicate of QC samples at high QC (4 μg/mL) level. The peak area ratio between total GB3 (six isoforms) and ISTD was used for the stability evaluation. GB3 in mouse plasma and tissue QC samples were stable for at least 3 h at room temperature and three freeze-thaw cycles and at least two weeks of stability while storage at −20 °C with a precision value (RSD (%)) within 30% and the mean accuracy within ±30%.

### 3.3. Application for Animal Study

**Figure 6:**
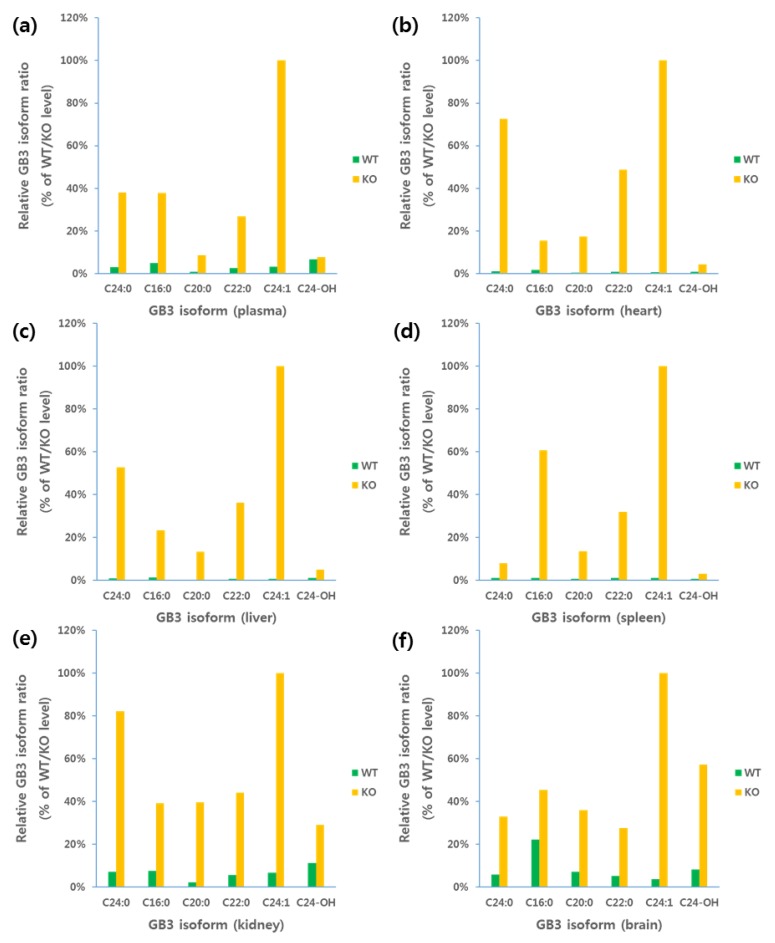
Relative GB3 isoform ratio in plasma and each of the tissues: (**a**) plasma; (**b**) heart; (**c**) liver; (**d**) spleen; (**e**) kidney; and (**f**) brain.

**Figure 7:**
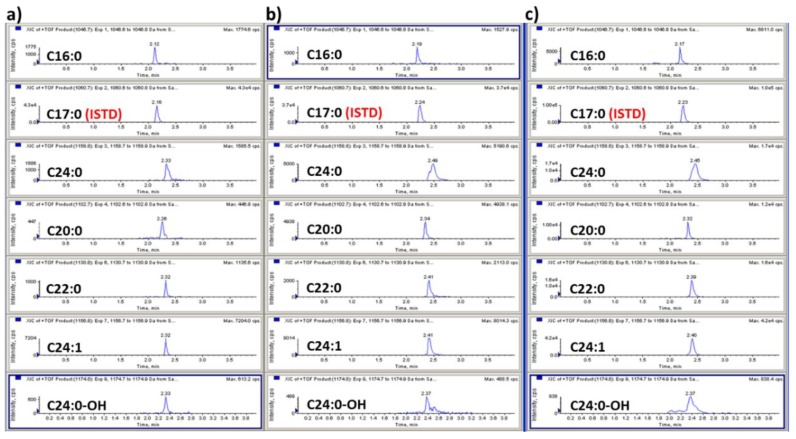
LC-MS chromatograms of GB3 in (**a**) plasma; (**b**) heart; and (**c**) spleen from the Fabry disease model mouse.

This LC-QTOF-MS/MS method was successfully applied for the quantification of GB3 in mouse plasma and tissue homogenate samples. We compared the average GB3 level in wild-type (WT, ICR strain) mouse (*n* = 12) to B6;129-Gla^tm1Kul^/J (*α-Gal A* Knock Out (KO)) mouse (*n* = 12) and the results are shown in Figure 6. The results show that GB3 accumulates significantly in the Fabry model mouse (plasma: 4.85–5.35 μg/mL; heart: 2.39–3.13 μg/mg; liver: 2.53–3.58 μg/mg; kidney: 6.81–7.05 μg/mg; spleen: 8.77–14.37 μg/mg; brain: 0.36–0.54 μg/mg) when compared to that in the ICR mice tested in this experiment (plasma: 19–21 ng/mL; heart: 3.16–4.14 ng/mg; liver: 3.05–4.32 ng/mg; kidney: 69.0–71.5 ng/mg; spleen: 4.74–7.76 ng/mg; brain: 4.70–7.05 ng/mg). Since this Fabry model mouse B6;129-Gla^tm1Kul^/J was newly developed in our lab, this was the first time to report the various GB3 isoform ratios in this mouse model. A supplementary LC-MS chromatogram of GB3 in plasma and tissues are also shown in Figure 7.

**Figure 8:**
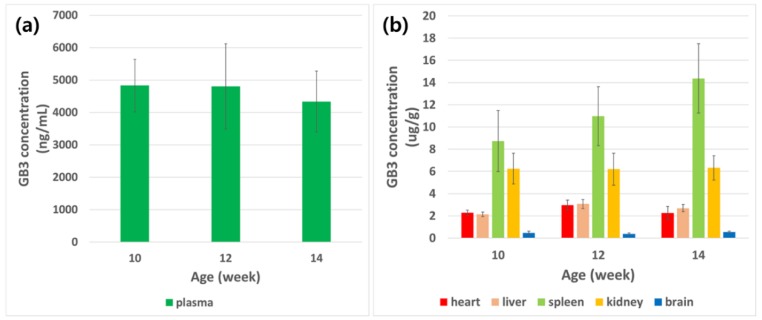
Age-related GB3 level changes in (**a**) plasma, and (**b**) tissues.

The age-related GB3 level change was also monitored in this study and the results are shown in Figure 8. In general, no significant increase of GB3 was observed in plasma, heart, liver, kidney, and brain. However, approximately 63.9% increase was observed in the spleen over a four week period (aged 10 to 14 weeks). This would be an important factor to consider when designing the study protocol in the future.

## 4. Discussion

During the early stage of Fabry disease drug discovery, it is very critical to screen the drug candidates with decent throughput. Since GB3 is an excellent biomarker which can represent the stage of Fabry disease in the Fabry model mice, we have developed a simple and robust bioanalytical method for the quantitation of GB3, particularly for the Fabry model mouse B6;129-Gla^tm1Kul^/J for the first time. This method also met our throughput criteria (>300 plasma and tissue samples per day) which were sufficient enough to cover the compounds in the screening stage. 

The calibration curve was produced in plasma as well as each of the tissues separately with a final concentration of the calibration curve range was 0.042–10 μg/mL for GB3 in plasma and 0.082–20 μg/g for GB3 in tissues, respectively. For assay performance, the within-run accuracy met the acceptance criteria of ±30% which ranged from 75 to 110% with precision values ≤30% for plasma and tissue samples, which is acceptable for early drug discovery. A preliminary stability test including short-term (1, 2, and 3 h), freeze-thaw (three cycles), and long-term stability (one and two weeks) show the precision and accuracy values within the acceptance criteria of ±30%.

The assay was also applied for the quantification of GB3 in Fabry model mice (B6;129-Gla^tm1Kul^/J) and wild-type mice (ICR strain) for the first time. The result shows that GB3 accumulates significantly in the Fabry model mouse with a relative difference in isoform ratios for each of the organs. There was no meaningful alteration of the GB3 level dependent to disease progression in plasma, heart, liver, kidney, and brain. However, GB3 level in spleen shows a meaningful age-related increase (>60% increase in four weeks) of GB3 level. Due to this method, we were able to screen many Fabry drug candidates for their efficacy of lowering GB3 levels in various tissues, as well as in plasma samples. This was particularly very important to evaluate the drug candidate’s efficacy in brain which is extremely critical for the drug candidate’s efficacy from the central nerve systems’ perspectives.

In conclusion, this rapid LC-QTOF-MS/MS method was very useful for the screening of drug candidates to evaluate their in vivo efficacy, as well as biomarker studies in the Fabry model mice.

## Figures and Tables

**Table 1:** Mass spectrometry conditions for six Globotriaosylceramide (GB3) isoforms.

**TOF-MS Condition**			
**GS1**	50	**CUR (Curtain Gas)**	30
**GS2**	50	**ISVP (Ion Spray Voltage)**	5500
**SRM High Sensitive Scan Mode, Positive**			
**GB3 Isoform**	**Parent-To-Parent Transition**	**Declustering Voltage (DP)**	**Collision Energy (CE)**
C16:0-GB3	1046.7→1046.7	100	66
C17:0-GB3	1060.7→1060.7 (ISTD)	100	66
C20:0-GB3	1102.7→1102.7	100	66
C22:0-GB3	1130.8→1130.8	100	66
C24:1-GB3	1156.8→1156.8	100	66
C24:0-GB3	1158.8→1158.8	100	66
C24:0-OH-GB3	1174.8→1174.8	100	66

**Table 2:** Quality control results and statistics from the qualification run for Globotriaosylceramide (GB3).

Matrix	QC Samples	Mean Concentration (ng/mL)	RSD (%)	Mean Accuracy (%)	*n*
**Plasma**	QC medium (400 ng/mL)	314.28	15.06	78.57	3
	QC high (2000 ng/mL)	2131.34	5.92	106.57	3
**Heart**	QC medium (800 ng/mL)	779.48	11.96	99.02	3
	QC high (4000 ng/mL)	3286.93	8.41	83.23	3
**Liver**	QC medium (800 ng/mL)	823.21	14.53	102.90	3
	QC high (4000 ng/mL)	3395.26	8.66	84.88	3
**Spleen**	QC medium (800 ng/mL)	848.22	10.64	106.03	3
	QC high (4000 ng/mL)	3861.45	25.49	96.54	3
**Kidney**	QC medium (800 ng/mL)	839.15	26.16	104.89	3
	QC high (4000 ng/mL)	4275.53	9.38	106.89	3
**Brain**	QC medium (800 ng/mL)	740.97	11.60	92.62	3
	QC high (4000 ng/mL)	3826.03	15.16	95.65	3

**Table 3:** Short-term stability results for GB3.

Matrix	Time Point (min)	Mean Area Ratio	RSD (%)	Mean Accuracy (%)	*n*
**Plasma**	0	3.78	6.54	100.00	3
	60	3.27	6.02	86.66	
	120	3.64	9.04	96.49	
	180	4.12	13.53	109.20	
**Heart**	0	3.06	12.10	100.00	3
	60	3.09	1.70	101.09	
	120	2.88	9.98	94.34	
	180	2.53	12.23	82.83	
**Liver**	0	3.14	9.73	100.00	3
	60	3.07	12.30	97.80	
	120	3.17	12.58	100.97	
	180	3.01	3.92	95.85	
**Spleen**	0	3.51	8.45	100.00	3
	60	2.89	8.54	82.31	
	120	3.02	11.61	86.14	
	180	2.50	3.18	71.32	
**Kidney**	0	2.42	10.26	100.00	3
	60	3.03	2.88	125.16	
	120	2.95	14.21	121.72	
	180	2.69	5.63	111.00	
**Brain**	0	1.48	3.70	100.00	3
	60	1.37	16.01	92.77	
	120	1.37	16.97	92.42	
	180	1.55	16.97	105.07	

**Table 4:** Freeze-thaw stability results for GB3.

Matrix	Control/FT-3 Cycle	Mean Area Ratio	RSD (%)	Mean Accuracy (%)	*n*
**Plasma**	Control	2.31	1.91	100.00	3
	FT-3 cycle	2.24	7.97	97.14	
**Heart**	Control	2.27	11.81	100.00	3
	FT-3 cycle	2.19	7.70	96.63	
**Liver**	Control	1.93	1.43	100.00	3
	FT-3 cycle	1.97	21.40	102.16	
**Spleen**	Control	2.23	6.15	100.00	3
	FT-3 cycle	2.12	10.07	95.20	
**Kidney**	Control	2.14	5.96	100.00	3
	FT-3 cycle	2.11	14.91	98.69	
**Brain**	Control	1.34	7.09	100.00	3
	FT-3 cycle	1.32	5.68	98.21	

**Table 5:** Long-term stability results for GB3.

Organ	Week	Mean Area Ratio	RSD (%)	Mean Accuracy (%)	*n*
**Plasma**	0 week	2.26	3.36	100	3
	1 week	2.29	14.55	101.46	3
	2 week	2.27	11.23	100.23	3
**Heart**	0 week	3.62	17.56	100	3
	1 week	3.81	8.19	105.32	3
	2 week	3.94	12.43	108.84	3
**Liver**	0 week	2.76	12.83	100	3
	1 week	2.52	0.73	91.18	3
	2 week	3.08	6.72	111.51	3
**Spleen**	0 week	4.05	4.44	100	3
	1 week	3.73	15.32	91.91	3
	2 week	3.72	5.13	91.8	3
**Kidney**	0 week	2.63	14.47	100	3
	1 week	2.69	10.44	102.31	3
	2 week	2.77	11.44	105.4	3
**Brain**	0 week	0.82	10.86	100	3
	1 week	0.8	7.71	97.46	3
	2 week	0.77	21.72	93.86	3
